# E-cigarette and food flavoring diacetyl alters airway cell morphology, inflammatory and antiviral response, and susceptibility to SARS-CoV-2

**DOI:** 10.1038/s41420-022-00855-3

**Published:** 2022-02-15

**Authors:** Stephanie N. Langel, Francine L. Kelly, David M. Brass, Andrew E. Nagler, Dylan Carmack, Joshua J. Tu, Tatianna Travieso, Ria Goswami, Sallie R. Permar, Maria Blasi, Scott M. Palmer

**Affiliations:** 1 Duke Center for Human Systems Immunology and Department of Surgery, Durham, NC USA; 2 Duke Clinical Research Institute and Department of Medicine, Duke University Medical Center, Durham, NC USA; 3 Duke Human Vaccine Institute, Duke University Medical Center, Durham, NC USA; 4 Department of Pediatrics, Duke University Medical Center, Durham, NC USA; 5 Department of Medicine, Division of Infectious Diseases, Duke University Medical Center, Durham, NC USA; 6 Department of Pediatrics, Weill Cornell Medicine, New York, NY USA

**Keywords:** Respiratory tract diseases, Infectious diseases

## Abstract

Diacetyl (DA) is an α-diketone that is used to flavor microwave popcorn, coffee, and e-cigarettes. Occupational exposure to high levels of DA causes impaired lung function and obstructive airway disease. Additionally, lower levels of DA exposure dampen host defenses in vitro. Understanding DA’s impact on lung epithelium is important for delineating exposure risk on lung health. In this study, we assessed the impact of DA on normal human bronchial epithelial cell (NHBEC) morphology, transcriptional profiles, and susceptibility to SARS-CoV-2 infection. Transcriptomic analysis demonstrated cilia dysregulation, an increase in hypoxia and sterile inflammation associated pathways, and decreased expression of interferon-stimulated genes after DA exposure. Additionally, DA exposure resulted in cilia loss and increased hyaluronan production. After SARS-CoV-2 infection, both genomic and subgenomic SARS-CoV-2 RNA were increased in DA vapor- compared to vehicle-exposed NHBECs. This work suggests that transcriptomic and physiologic changes induced by DA vapor exposure damage cilia and increase host susceptibility to SARS-CoV-2.

## Introduction

Diacetyl (DA) is a volatile α-diketone used to impart a buttery-like aroma and flavor to a variety of food products and electronic cigarettes (e-cigs). Despite the widespread use of flavoring chemicals in e-cigs [1], very little is known regarding the negative impact of these chemicals on e-cig users. Although concerns regarding e-cig use and vaping primarily focus on nicotine and cannabis-derived compounds like tetrahydrocannabinol, DA is linked to a variety of toxic effects in the lung and airway cells. For example, DA exposure promotes oxidative stress, upregulates inflammatory processes including IL-8 secretion, cilia loss, and dedifferentiation of the epithelial layer in vitro [2–6]. This is relevant considering clinical and experimental evidence links the development of bronchiolitis obliterans (“popcorn lung”) and irreversible lung disease to occupational DA exposure [7].

The significant proportion of e-cig use among youth combined with the demonstrated adverse effects of e-cig components like DA on airway epithelium, suggests e-cig use may be an important risk factor for coronavirus disease (COVID)-19, particularly in adolescents. Indeed, COVID-19 diagnosis was five times more likely among adolescent and young adult e-cig users as compared to non-users [8]. Previous evidence demonstrated that e-cig exposure significantly impaired antiviral host defenses and increased the influx of inflammatory neutrophils, potentiating immune-based pathology [9, 10]. Additionally, chronic e-cigarette vapor exposure has been shown to alter the physiology of lung epithelial cells and resident immune cells and to promote poor response to viral challenge in mice [11]. The broad range of pulmonary toxicity of e-cig components [12, 13], and the impact of flavoring agents such as DA on the airway epithelium inflammatory and antiviral responses, may predispose individuals to lung injury and severe COVID-19 following SARS-CoV-2 infection.

To evaluate the impact of multiple DA exposures on human airway epithelium, we assessed cellular morphology and transcriptional profiles of DA vapor-exposed normal human bronchial epithelial cells (NHBECs). Additionally, we determined if DA vapor exposure altered susceptibility to SARS-CoV-2 infection. This work provides new insights into early transcriptional responses and cilia loss to repeated DA vapor exposure that may contribute to the development of flavoring-induced airway disease and increased susceptibility to SARS-CoV-2 infection.

## Results

### DA vapor exposure drives differential transcriptomic responses in NHBECs and reveals cilia injury signatures

**Fig. 1:**
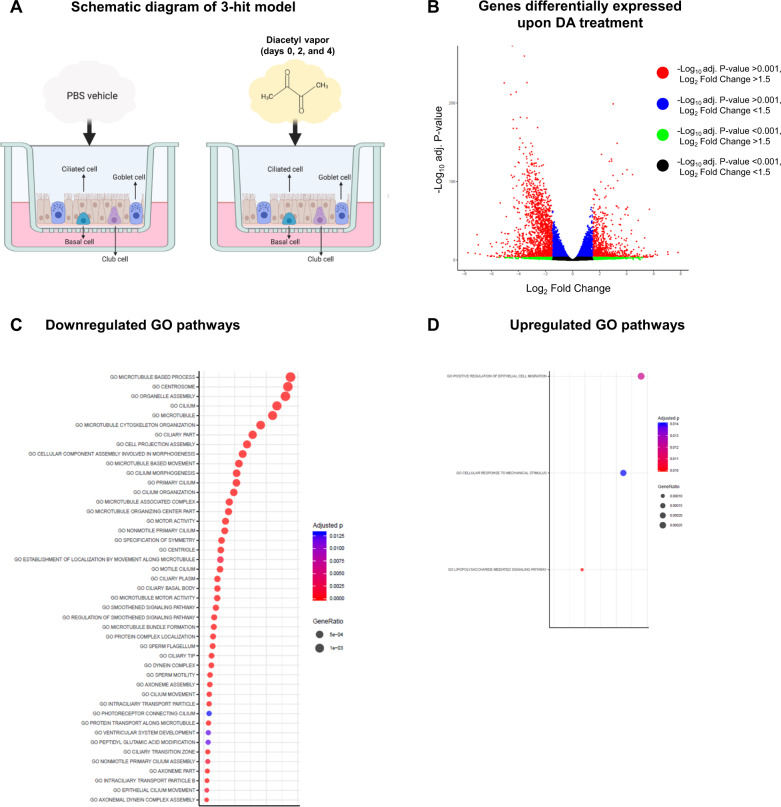
Diacetyl (DA) vapor exposure of normal human bronchial epithelial cells (NHBECs) drives differential transcriptomic responses. **A** Schematic diagram of 3-hit model of PBS vehicle- and diacetyl (DA) vapor exposure of NHBECs. Triplicate wells of NHBECs from donor TBE-20 were exposed to PBS vehicle or DA vapor at 0, 2, and 4 days prior to RNA-seq analysis on day 6. **B** Volcano plot of RNA-seq results where genes are represented by red dots (−log_10_ adjusted *P* value < 0.001, log_2_ fold change > 1.5), blue dots (−log_10_ adjusted *P* value < 0.001, log_2_ fold change < 1.5), green dots (−log_10_ adjusted *P* value > 0.001, log_2_ fold change > 1.5), and black dots (−log_10_ adjusted *P* value > 0.001, log_2_ fold change < 1.5). **C** Down- and **D** upregulated gene ontology (GO) pathways with an FWER adjusted *P* value < 0.015 were reported. Dot size represents gene ratio and color schema represents FWER adjusted *P* values.

**Fig. 2:**
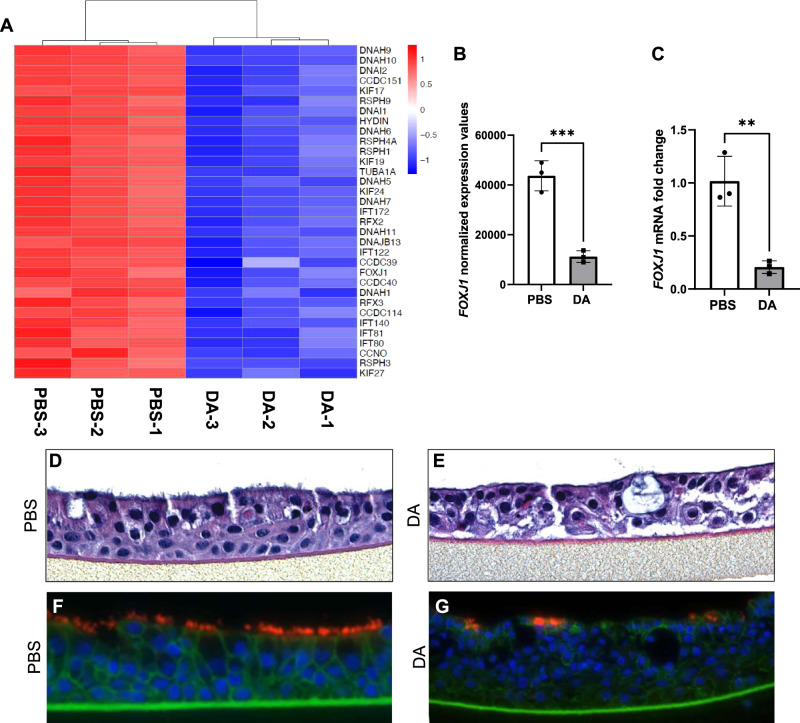
Decreased expression of genes associated with cilia structure and motility in normal human bronchial epithelial cells (NHBECs) treated with diacetyl (DA) vapor. **A** Heatmap of DEGs that are downregulated following DA treatment and associated with cilia structure and motility. Red represents relative upregulation of gene expression and blue represents relative downregulation of gene expression. Genes are arranged by log_2_ fold change with the largest log_2_ fold change at the top and the smallest log_2_ fold change at the bottom. **B** *FOXJ1* normalized expression data from RNA-seq analysis. **C** Validation of *FOXJ1* expression by quantitative real-time polymerase chain reaction (qRT-PCR) shown as fold change after DA vapor exposure. **D** Pathological assessment after hematoxylin and eosin (H&E) staining of PBS vehicle and **E** DA vapor-exposed NHBECs. **F** Assessment of acetylated tubulin (red, Alex Fluor 594), β-catenin (green, Alex Fluor 488) and nuclei (blue, DAPI) expression in NHBECs via immunofluorescent staining after DA vapor or **G** PBS vehicle exposure. All quantified results are expressed as mean ± SD. *n* = 3 per group. Significance was determined by an unpaired *t* test. ****P < 0.0001, ***P < 0.001.

NHBECs were exposed to PBS vehicle or DA vapor (Fig. 1A) for 1 h on days 0, 2, and 4 prior to RNA isolation and RNA-seq analysis on day 6. The hierarchical and principal component analyses and heatmap (Fig. S1A–C) of the differentially expressed genes (DEGs) demonstrate that DA vapor altered transcriptomic expression in NHBECs. To visualize the quantitative effects of DA vapor exposure in NHBECs, a volcano plot was generated in which the log_2_ fold change was plotted as a function of the negative log-transformed adjusted *P* value for all DEGs (Fig. 1B). To identify gene ontology (GO) and hallmark gene sets associated with altered expression after DA vapor exposure, gene set enrichment analysis (GSEA) was performed (1). We identified forty-five (Fig. 1C) and three (Fig. 1D) GO pathways that were significantly enriched among down- and upregulated genes (using a stringent cutoff of FWER adjusted *P* value ≤ 0.01) after DA vapor exposure, respectively. Within the downregulated GO pathways (Table S1), we identified multiple genes that are required for cilia structure and motility (Fig. 2A, Table S2). These included proteins involved in the structure of the axoneme including dynein axonemal heavy chains (DNAHs), dynein cytoplasmic 2 heavy chains, dynein axonemal intermediate chains, and several transcripts for radial spokehead proteins (Fig. 2A, Table S2) [14]. Additionally, intraflagellar transport (IFT) genes, required for maintenance and formation of cilia, and kinesin family member (KIF) genes, necessary for proper cilia structure, motility and length, were highly enriched in these downregulated GO pathways (Fig. 2A, Table S2) [15]. The transcription factor Forkhead box J1 (*FOXJ1*), required for multiciliated cell differentiation, along with transcriptional coactivators regulatory factor X (*RFX*)*2* and *RFX3* [16] were also overrepresented in this analysis. We confirmed *FOXJ1* mRNA expression was significantly decreased in DA vapor—compared to PBS vehicle-exposed NHBECs via qRT-PCR (Fig. 2B, C). Assessment of hematoxylin and eosin staining in NHBECs demonstrated loss of cilia and flat dysplastic epithelium after DA vapor—compared to PBS vehicle exposure (Fig. 2D, E). Immunofluorescent staining of NHBECs revealed decreased expression of acetylated tubulin after DA vapor- compared to PBS vehicle exposure (Fig. 2F, G), further validating the transcriptomic data demonstrating broad suppression of ciliogenesis after DA exposure.

### DA vapor exposure increased CD14 and IL1B gene expression, HA production and promoted mobilization of NHBECs

**Fig. 3:**
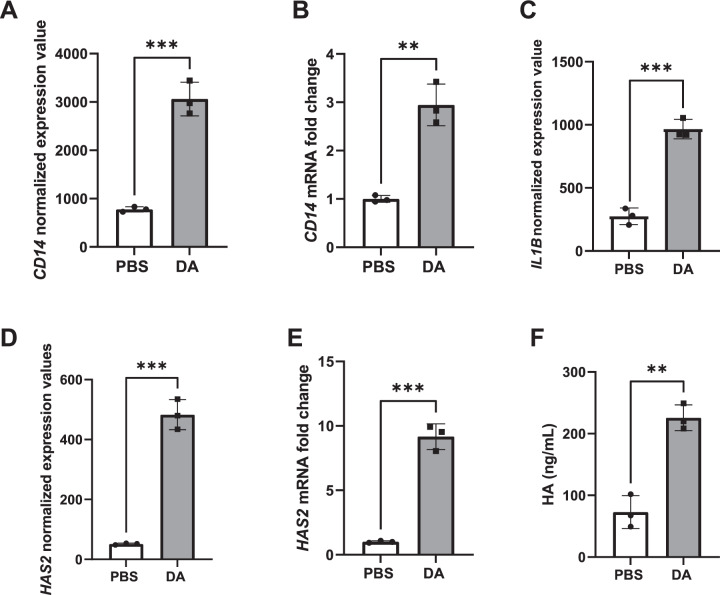
DA vapor exposure increased *CD14*, *IL1B*, and hyalruonan synthase (*HAS2*) transcript expression as well as hyalruonan (HA) production in normal human bronchial epithelial cells (NHBECs). **A** *CD14* normalized expression data from RNA-seq analysis and **B** validation of *CD14* expression by quantitative real-time polymerase chain reaction (qRT-PCR) shown as fold change after PBS or DA vapor exposure. **C** *IL1B* normalized expression data from RNA-seq analysis. **D** *HAS2* normalized expression data from RNA-seq analysis and **E** validation of *HAS2* expression by qRT-PCR shown as fold change after PBS or DA vapor exposure. **F** Quantification of HA production in the NHBEC basal medium via ELISA. All quantified results are expressed as mean ± SD. *n* = 3 per group. Significance was determined by an unpaired *t* test. ***P < 0.001, **P < 0.01.

**Fig. 4:**
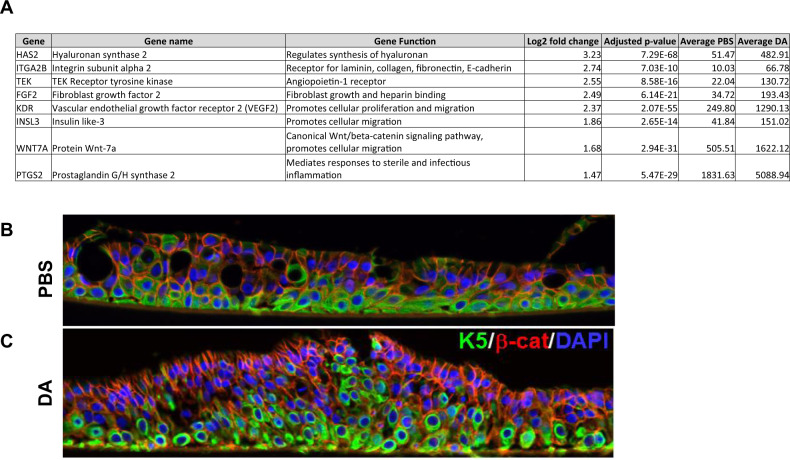
DA vapor exposure induces an epithelial cell mobilization phenotype. **A** Upregulated genes from the *Positive regulation of epithelial cell migration* gene ontology pathway that fell within the stringent cutoff of our dataset (FWER adjusted *P* value < 0.001, log_2_ fold change >1.45) **B** Representative confocal images of keratin 5 (K5, green, Alex Fluor 488), β-catenin (green, Alex Fluor 59) and nuclei (blue, DAPI) expression in NHBECs via immunofluorescent staining after PBS vehicle or **C** DA vapor exposure.

Three of the most significantly upregulated GO pathways in response to DA exposure included Lipopolysaccharide (LPS) mediated signaling, Positive regulation of epithelial cell migration and Cellular response to mechanical stimulus. Within these GO pathways, we identified upregulated genes that fell within the stringent cutoff of our dataset (FWER adjusted *P* value < 0.001, log_2_ fold change > 1.5) (Table S3). Among these, included lymphocyte antigen 96 (LY96) [also known as myeloid differentiation factor 2 (MD-2) encoding protein MD2] and CD14, both associated with TLR4-mediated LPS signaling [17] within the LPS mediated signaling GO pathway. Further validation by qRT-PCR indicated significant upregulation of CD14 mRNA in DA vapor- compared to PBS vehicle-exposed NHBECs, which corresponded to the normalized expression values from the RNA-seq analysis (Fig. 3A, B). IL1B, an inflammatory cytokine found in multiple upregulated GO pathways was significantly upregulated in DA vapor- compared to PBS vehicle-exposed cells (Fig. 3C). Within the Positive regulation of epithelial cell migration GO pathway, HAS2 was significantly upregulated gene both by normalized expression as determined by RNA-seq analysis (Fig. 3C) and mRNA levels from qRT-PCR (Fig. 3D). Notably, hyalruonan (HA) production in the NHBEC supernatant was also significantly increased after DA vapor rather than PBS vehicle exposure (Fig. 3E), further validating the HAS2 transcript data. Other transcripts associated with the Positive regulation of epithelial cell migration GO pathway including ITGA2, VEGF2, WNT7A and PTGS2 were expressed at high levels in NHBECs and were significantly higher after DA vapor compared to PBS vehicle exposure (Fig. 4A). Additionally, immunofluorescence staining demonstrates relocalization of basal cells throughout the pseudostratified epithelium in DA vapor- (Fig. 4C) compared to PBS vehicle-exposed (Fig. 4B) NHBECs.

### Hallmark gene set analysis reveals pathways associated with interferon-alpha responses and DNA damage repair are downregulated while pathways associated with sterile inflammation are upregulated in NHBECs after DA vapor exposure

To further characterize transcriptional profiles, we applied the pre-ranked GSEA to 50 Hallmark gene sets with well-defined biological mechanisms and coherent expression levels. The Hallmark gene set analysis demonstrated that exposure to DA vapor resulted in enriched pathways amongst down- (Fig. S2A) and upregulated (Fig. S2B) genes, respectively. Similar to the GO pathways, Hallmark pathway analysis revealed a decrease in genes associated with interferon-alpha responses. Additionally, genes associated with E2F targets and the G2/M checkpoint associated with DNA replication and DNA damage repair, respectively, were downregulated after DA vapor exposure (Fig. S2A). Particularly relevant to DA-induced pattern recognition receptor (PRR) responses observed in the upregulated GO pathway analysis were Hallmark pathways associated with sterile inflammation (Table S4) including the *TNF-α signaling* via *NF-κB, Hypoxia*, *Inflammatory response*, and *IL-6 JAK STAT3 signaling* gene sets. Additionally, hypoxia induces p53 which mediates cellular apoptosis [18], both of which were upregulated in the Hallmark gene set analysis (i.e., *P53 pathway* and *Apoptosis*). *Kras* and *TGF-β signaling* pathways associated with repair were also included in the significantly upregulated gene sets after Hallmark gene set analysis. Overall, Hallmark pathways associated with sterile inflammation and repair were both upregulated after DA exposure.

### Decreased expression of multiple ISGs after DA vapor exposure

**Fig. 5:**
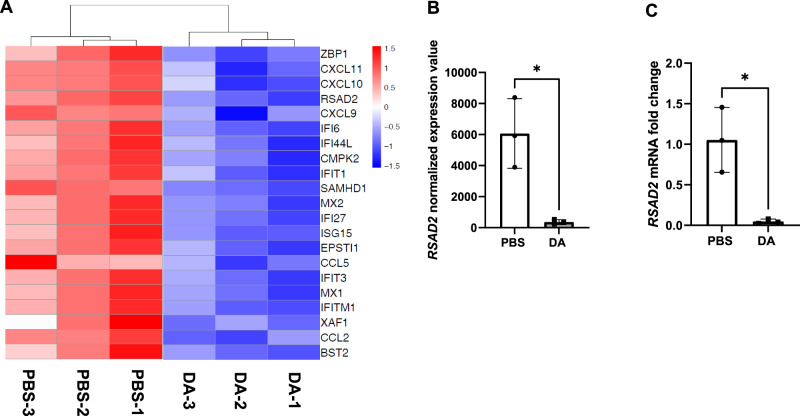
Decreased expression of interferon-stimulated genes after diacetyl (DA) vapor exposure in normal human bronchial epithelial cells (NHBECs). **A** Heatmap of interferon-stimulated genes that are downregulated following DA treatment. Red represents relative upregulation of gene expression and blue represents relative downregulation of gene expression. Genes are arranged by log_2_ fold change with the largest log_2_ fold change at the top and the smallest log_2_ fold change at the bottom. **B** RSAD2 normalized expression data from RNA-seq analysis and **C** validation of RSAD2 expression by qRT-PCR shown as fold change after DA vapor exposure. All quantified results are expressed as mean ± SD. *n* = 3 per group. Significance was determined by an unpaired *t* test. *P < 0.05.

In both the GO and Hallmark gene set pathway analyses, interferon responses were enriched among the downregulated pathways. To further evaluate the effect of DA vapor exposure on innate immune factors important in antiviral protection of epithelial cells, we identified DEGs of the interferon stimulated gene (ISG) family (FWER adjusted *P* value ≤ 0.01) after DA vapor exposure (Fig. 5A, Table S5). DA vapor exposure in NHBECs downregulated expression of ISGs previously demonstrated to suppress replication of both RNA and DNA viruses (Fig. 5A) [19]. Radical SAM domain-containing 2 [RSAD2 (also known as Viperin)], a potent antiviral ISG, was one of the most negatively DEG impacted by DA vapor exposure. RSAD2 mRNA expression was further validated by qRT-PCR was found to be significantly lower in DA vapor-compared to PBS vehicle-exposed NHBECs, which corresponded to normalized expression values from the RNA-seq analysis (Fig. 5B, C). Additionally, multiple chemotactic factors involved in the recruitment of immune cells were significantly downregulated (Table S5). The suppression of multiple ISGs and chemotactic factors after DA vapor exposure suggests increased susceptibility to viral infection in NHBECs.

### DA vapor exposure increases susceptibility of NHBECs to SARS-CoV-2 infection

**Fig. 6:**
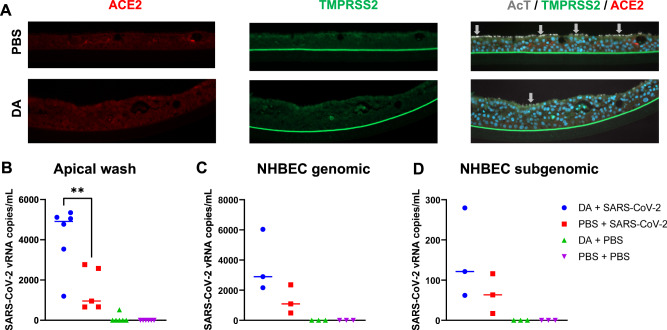
DA treatment does not alter ACE2 receptor or TMPRSS2 cellular protease expression but increases normal human bronchial epithelial cell (NHBEC) susceptibility to SARS-CoV-2 infection. **A** Immunofluorescence was performed on cross sections of formalin-fixed and paraffin-embedded NHBECs to visualize ACE2 (red, Alex Fluor 594), TMPRRS2 (green, Alex Fluor 488), acetylated tubulin (AcT, gray), and nuclei (blue, DAPI). Images are captured at 20X magnification. Total SARS-CoV-2 RNA levels were determined in **B** apical wash and **C** genomic and **D** subgenomic viral RNA levels were determined in NHBECs. Data points represent replicates from one NHBEC donor. Significance was determined by a one-way ANOVA with Tukey’s corrections for multiple comparisons. **P < 0.01.

We first determined if DA vapor exposure alters expression of SARS-CoV-2 receptor ACE2 and transmembrane serine protease 2 (TMPRSS2), a cellular protease required for SARS-CoV-2 entry. Despite the negative impact of DA on NHBEC morphology and decrease in cilia, IHC staining confirmed that DA vapor did not ameliorate ACE2 or TMPRRS2 expression (Fig. 6A), suggesting that SARS-CoV-2 could still bind and enter DA vapor-exposed NHBECs. To determine the impact of DA vapor exposure on SARS-CoV-2 susceptibility, NHBECs were first exposed to PBS vehicle or DA vapor for 1 h on days 0, 2, and 4, and infected with SARS-CoV-2 USA-WA1/2020 (MOI of 0.5), after 24 h of the final DA exposure. As measures of viral infection and replication, we determined both cell-free (Fig. 6B) and cell-associated (Fig. 6C, D) viral RNA levels after 48 h of infection. DA vapor exposure significantly increased viral replication in NHBECs, as indicated by total viral RNA release in apical media (Fig. 6B) and genomic (Fig. 6C) and subgenomic (Fig. 6D) viral RNA levels. SARS-CoV-2 infection did not further exacerbate cell dysplasia or cilia loss in DA vapor-exposed NHBECs (Fig. S3). Our data indicated an increased susceptibility of NHBECs to SARS-CoV-2 infection.

## Discussion

Damage to airway epithelium caused by cigarette smoking, environmental pollutants, and pathogenic infections, amongst others, is associated with cilia loss and inflammation in the airway epithelium [20–23]. Emerging evidence also suggests that these factors increase susceptibility to SARS-CoV-2 infection and COVID-19 [24–28]. Whether DA also impacts susceptibility to SARS-CoV-2 infection and/or COVID-19 severity is not well established. Recent studies suggest that flavoring chemicals like DA have direct toxicity to airway cells and could contribute to adverse health outcomes [29–31]. Specifically, DA vapor exposure causes loss of epithelial resistance, flattening of the airway epithelium, and loss of cilia in both in vitro and in vivo models [32].

Airway epithelium contain motile cilia which are an integral part of the mucociliary escalator, removing fluids, mucins, and particulates from the airway [33, 34]. Previous evidence demonstrates that expression of multiple genes involved in cilia biogenesis are significantly downregulated in NHBECs when DA liquid was added to cell culture media [29]. Similarly, we observed loss of cilia on the surface of NHBECs and broad suppression of transcripts related to cilia structure and function after DA vapor exposure. For example, *FOXJ1*, a master regulator of motile ciliogenesis [35, 36], and *RFX2* and *RFX3*, transcriptional coactivators of FoxJ1 [16, 37], were significantly downregulated in our RNA-seq analysis. Considering cilia assembly is under strict transcriptional control, DA-induced suppression of these transcripts could result in an overall decrease in the cell’s cilia production program. Other transcripts overrepresented in the top 10 downregulated pathways were associated with the radial spoke and spokehead, dynein proteins, IFT proteins KIF member genes and *CCNO*, *HYDIN* and *TUBA1A* transcripts. We further validated the transcriptomic data and observed loss of cilia by cell histology and decreased protein expression of acetylated tubulin. Acetylation of α-tubulin marks stable, long-lived subpopulations of cilia and flagella, and decreased acetylated tubulin indicates primary cilia loss [38]. The suppression of cilia-related genes in addition to cilia loss is likely to result in decreased mucociliary clearance and increased cellular susceptibility to pathogens. Indeed, genetic defects in *DNAH5, HYDIN,* and *TUBA1A* and others result in primary cilia dyskinesia (PCD); a variety of clinical manifestations including ineffective mucociliary clearance and recurrent lower respiratory tract infections [39–48].

Various endogenous danger-associated molecular patterns (DAMPs) are produced after non-pathogenic cellular injury that activate PRRs and induce sterile inflammation [49]. HA is an extracellular matrix glycosaminoglycan released after sterile injury or pathogenic infection and exerts size-dependent effects. For example, low molecular weight (LMW) HA acts as a proinflammatory DAMP via toll-like receptor (TLR) signaling [50, 51]. We demonstrated that DA exposure increased *HAS2* transcript expression and HA production in NHBECs. While we did not measure LMW HA specifically, we observed an increase in transcripts associated with PRR signaling. For example, *CD14* and *LY96* gene expression were significantly upregulated after DA treatment. Membrane-bound CD14 is a PRR that recognizes diverse microbial products [52–56] and LY96 (MD-2) physically associates with TLR4 to mediate LPS signaling via CD14 [17]. Interestingly, HA is demonstrated to signal through a unique complex of TLR4, MD-2 and CD44 [57]. While HA has not been demonstrated to signal through CD14 directly, the interaction of HA binding proteoglycans like versican and CD14 can stimulate downstream cellular responses [58–60]. Additionally, HA exudates have been observed in the lungs of deceased COVID-19 patients [61, 62], suggesting a role for this molecule in acute respiratory distress syndrome caused by SARS-CoV-2. We also observed mobilization of NHBECs after DA vapor exposure. After mechanical damage to airway epithelium, epithelial cell migration is integral to tissue repair [63]. Previous studies demonstrate that rats that inhale DA exhibit ultrastructural changes in the trachea consistent with epithelial spreading and migration [64]. Interestingly, increased basal cell mobilization is also observed during SARS-CoV-2 infection in NHBECs in vitro [65]. Altogether these results suggest that damage to the airway epithelium, whether by toxin exposure or viral infection, results in epithelial cell mobilization that will likely impact the processes involved in epithelium repair.

We identified downregulated Hallmark pathways associated with DNA replication and DNA damage repair after DA exposure. This is consistent with previous literature demonstrating DA covalently binds to guanyl nucleotides leading to DNA unwinding and cellular apoptosis [66]. Damage is likely further exacerbated by an increase in DA-induced hypoxia and sterile inflammation as indicated in our Hallmark pathway analysis. Recent studies have demonstrated a link between hypoxia and sterile inflammation via IL-1β priming of the NLRP3 inflammasome [67–71]. The upregulation of the *Hypoxia* and *Inflammatory response* Hallmark pathways and significant increase in the *IL1B* gene observed in our RNA-seq analysis suggests a similar mechanism after DA exposure in NHBECs. Hypoxia induces p53 which mediates cellular apoptosis [18], both of which are upregulated in the Hallmark gene set analysis (i.e. *P53 pathway* and *Apoptosis*). Furthermore, the upregulation of *TNF-α signaling* via *NF-κB* and *IL-6 JAK STAT3 signaling* Hallmark pathways highlight a potential role for TNF-α and IL-6 as triggers of sterile inflammation as previously demonstrated [72, 73]. The increase in *Kras* and *TGF-β signaling* pathways after DA vapor exposure suggests a role for TGF-β in mediating tissue remodeling after hypoxia-induced injury [74]. KRAS is a member of the Ras protein family, which functions as a signal transducer between epidermal growth factor receptor (EGFR) signaling and the MAPK pathway (Ras/MAPK pathway) [75]. Ras/MAPK activity induces p53 phosphorylation, enabling the interaction of p53 and TGF-β. Overall, the Hallmark gene set analysis suggests that DA vapor exposure results in changes in NHBECs similar to those observed in hypoxia leading to inflammation and compensatory TGF-β-mediated repair signatures.

We observed suppression of multiple ISG transcripts important in antiviral protection of epithelial cells after DA vapor exposure in NHBECs. ISG products exhibit many diverse functions but collectively are highly effective at resisting and controlling viral infections [76]. For example, RSAD2 (Viperin) was one of the most significantly decreased ISG after DA vapor exposure. Viperin has been demonstrated to inhibit replication of multiple respiratory viruses including measles, respiratory syncytial virus, and influenza [77–79]. Previous studies demonstrate that e-cig exposure significantly impaired antiviral host defenses in the lung [9, 10]. Ours is the first evidence that a flavoring chemical directly reduces expression of multiple ISG transcripts in NHBECs. These data demonstrate that DA suppressed the transcriptomic expression of important antiviral and immune factors that could lead to increased susceptibility to SARS-CoV-2.

The evidence that DA exposure induces a loss of cilia and acetylated tubulin combined with a decrease in transcriptomic expression of important ISGs led us to hypothesize that DA vapor-exposed NHBECs cells would be more susceptible to SARS-CoV-2 infection. Indeed, recent evidence demonstrates a loss of motile cilia and dedifferentiation of the epithelial cells after SARS-CoV-2 infection both in vitro and in vivo [65]. After confirming SARS-CoV-2 receptor ACE2 and cellular protease TMPRSS2 were still present after DA vapor exposure, we infected DA vapor and PBS vehicle-exposed NHBECs with SARS-CoV-2. The increase in the mean of both genomic and subgenomic SARS-CoV-2 RNA levels in NHBECs demonstrate that DA increases susceptibility of NHBECs to SARS-CoV-2 infection. These data are in line with a recent report demonstrating that direct cigarette smoke exposure reduces innate immune responses and increases the number of SARS-CoV-2 infected cells [25]. Additionally, this is consistent with other studies demonstrating negative impacts of e-cigs on the airway and host defenses. For example, mice exposed to e-cig vapor had altered lung lipid homeostasis and downregulated innate immune responses in alveolar macrophages and epithelial cells. When infected with influenza, e-cig vapor-exposed mice had increased lung inflammation and tissue damage [11]. Our data demonstrate that DA vapor induces cilia loss and specific pathological changes in the airway epithelium that increase susceptibility to viral infection, specifically against SARS-CoV-2, suggesting a potential mechanism for increased susceptibility in e-cigarette users.

## Methods

### EpiAirway tissues and vapor exposure

Fully differentiated human EpiAirway tissue inserts (0.6 cm^2^) were purchased from MatTek Corporation (Ashland, MA). EpiAirway tissues were generated from lower tracheal/bronchial cells (AIR-112-D2; TBE-20, (healthy, non-smoker, 13 yo male donor), and cultured at the air-liquid interface (ALI) at 37 °C (+5% CO_2_). Upon receipt, EpiAirway tissue inserts were placed into 1 mL of culture medium (MatTek) in 6 well culture plates for 16–18 h to equilibrate. The apical surface was then gently rinsed 2X with 0.4 mL PBS (MatTek) to remove excess mucus. The tissue inserts were transferred into 1 mL of fresh culture medium, which bathes the basolateral side of the tissue, and exposed to the apical surface to DA vapor for 1 h at 37 °C (+5% CO_2_) using vapor cups (MatTek). Specifically, 50 µl of PBS vehicle or 25 mM DA (104 µg/1100 ppm), was applied to a fiber pad in a vapor cup supplied by MatTek (AIR-1488 MILCEL-MTK-CAP-FP) which was then inverted and sealed (using silicone) on the tissue insert for 1 h, as previously described [4]. Tissue inserts were exposed to vehicle or DA vapor for 1 h on days 0, 2, and 4. The apical surface was rinsed twice with PBS prior to vapor exposure. The culture medium was collected every day and replaced with 1 mL of fresh medium. Basolateral supernatants were collected, centrifuged at 15,000 × *g* for 15 min at 4 °C to remove cellular debris, and stored at −80 °C until evaluation. Cells were washed, then frozen at −80 °C at day 6, 48 h after the last exposure, to preserve cells for future transcript analysis.

### RNA isolation and transcript analysis

Cells were lysed in 350 µl of Trizol, and RNA was extracted by phenol:chloroform method, and collected over RNAeasy column (Qiagen, Germantown, MD). Seven microliters of RNA was converted to cDNA using the high capacity reverse transcription kit (LifeTech, Carlsbad, CA). Transcripts were evaluated by TaqMan assay, where 40 ng of cDNA was used in each reaction with validated assays, and duplexed with β actin as the endogenous control. Ct values were determined using ABI 7500 RealTime PCR System (LifeTech, Carlsbad, CA) with SDS software version 1.5.1. Change in expression was calculated using the 2^−ΔΔCt^ method.

### HA ELISA

Clarified basolateral supernatants were evaluated for HA production using an HA ELISA (R&D Systems, Inc., Minneapolis, MN), following the manufacturer’s protocol.

### EpiAirway staining

EpiAirway tissue inserts were washed twice with 1× PBS, fixed with 10% formalin, and embedded in paraffin. Five micrometers sections were evaluated by immunofluorescence following deparaffinization and citrate buffer antigen retrieval. Sections were blocked with 5% BSA in PBS for 1 h at room temperature, primary and secondary antibodies were diluted in blocking solution and incubated overnight at 4 °C or room temperature for 90 min, respectively. Following secondary antibody, slides were washed in PBS and cover-slipped with DAPI in Fluoromount G mounting media (Southern Biotech, Birmingham, AL). Images were obtained using a Oberserver Z1 Zeiss microscope (Dublin, CA) equipped with a digital camera and processed using Zen-Pro (Dublin, CA). Primary antibodies used and their concentrations are as follow: mouse IgG1 anti-β catenin (1:400, BD, Franklin Lakes, New Jersey), mouse IgG2b anti-acetylated tublin (AcT) (1:10,000, Sigma, St Louis, MO), mouse IgG1 anti-TMPRSS2 (1:25, EMD Millipore, Temecula, CA), rabbit anti-ACE2 (1:50, Abcam, Cambridge, MA). Secondary antibodies used and their concentrations are as follows: Goat anti-IgG1 Alexa Fluor 488 (1:500, LifeTech, Carlsbad, CA), goat anti-IgG2b Alexa Fluor 594, 680 (1:500, LifeTech, Carlsbad, CA), donkey anti-rabbit Alexa Fluor 488 (1:500, LIfeTech, Carlsbad, CA)

### SARS-CoV-2 propagation and tittering

SARS-CoV-2 USA-WA1/2020 (BEI Resources) was propagated on Vero E6 cells (passage 1–2) at a multiplicity of infection (MOI) = 0.001 in virus diluent [DMEM supplemented with 2% FBS, 1× Penicillin/Streptomycin (Gibco), 1 mM sodium pyruvate (Gibco) and 1× NEAA (Gibco) at 37 °C in 5% CO_2_. At day 4 post infection, cell supernatant containing the released virus was harvested, centrifuged at 500 × *g* for 5 min, filtered through a 0.22 µM sterile vacuum filtration system, aliquoted and stored at −80 °C until further use.

Stock viral titer was determined by plaque assay. Briefly, 0.72 × 10^5^ Vero E6 cells were seeded in 6 well plates. The stock virus was serially diluted and incubated on cellular monolayer at 37 °C in 5% CO_2_. After 1 h, virus was aspirated, and cells were overlayed with carboxy-methyl cellulose (CMC) containing media 0.6% CMC, MEM supplemented with 1× Penicillin/Streptomycin (Gibco), 2% FBS, 1 mM sodium pyruvate (Gibco), 1× NEAA (Gibco), 0.3% sodium bicarbonate (Gibco), and 1× GlutaMAX (Gibco). After 4 days of incubation at 37 °C in 5% CO_2_, plaque assays were stained with 1% crystal violet in 10% neutral buffered formalin (NBF), and plaque forming unit/mL (PFU/mL) was determined.

### SARS-CoV-2 infection

One day after the third DA vapor exposure (day 5), the apical side of NHBECs cells were washed 1× with PBS and incubated with either SARS-CoV-2 at an MOI of 0.5 or PBS at 37 °C and 5% CO_2_, with intermittent plate rocking. At 48 h post SARS-CoV-2 inoculation, apical cell washes and cells were lysed in trizol for RNA isolation and viral analyses.

### qRT-PCR for detecting viral RNA

After 48 h of infection, NHBECs were resuspended in TRIzol reagent (Thermo Fisher). Cellular RNA was extracted by phase separation with chloroform and subsequently using the RNeasy Mini Kit (Qiagen). RNA from cell supernatants was extracted using the QIAamp viral RNA mini kit (Qiagen). A two-step qRT-PCR was used to detect viral RNA released in the cell supernatant. In the first step, viral cDNA for the nucleocapsid (N) gene was generated using SuperScript III Reverse Transcriptase (Invitrogen), following manufacturer’s instructions. For cDNA preparation of genomic and sub-genomic viral RNA, N-reverse primer (5′-GAGGAACGAGAAGAGGCTTG-3′) and N-forward primer (5′-CACATTGGCACCCGCAATC-3′) were used, respectively.

In the second step, 7 µl cDNA from step-1 was amplified using N gene forward primer (5′-CACATTGGCACCCGCAATC-3′), N gene reverse primer (5′-GAGGAACGAGAAGAGGCTTG-3′) and probe (5″-FAM-ACTTCCTCAAGGAACAACATTGCCA-QSY-3′) using Taqman mastermix (Thermo Fisher). The thermal cycling steps were: 50 °C for 2 min, 95 °C for 10 min, and 40 cycles of 95 °C for 15 s and 60 °C for 1 min, and qPCR was performed on a Step-One-Plus real-time PCR machine (Applied Biosystems) using the StepOne Software v2.3.

Viral RNA copy number/mL supernatant was assessed using pCDNA3.1(+)-N-eGFP plasmid (GenScript) as standard. We determined the copy number by using the following formula:

$${{{\mathrm{Number}}}}\,{{{\mathrm{of}}}}\,{{{\mathrm{copies}}}}\,{{{\mathrm{(molecules)}}}} = \frac{{{{{X}}}\;{{{\mathrm{ng}}}} \ast {{{\mathrm{6}}}}{{{\mathrm{.0221}}}} \times {{{\mathrm{10}}}}^{23}\;{{{\mathrm{molecules/mole}}}}}}{{\left( {{{{N}}} \ast {{{\mathrm{660}}}}\;{{{\mathrm{g}}}}/{{{\mathrm{mole}}}}} \right)^{\dagger} \ast 1 \times 10^9\;{{{\mathrm{ng}}}}/{{{\mathrm{g}}}}}}$$

and the following link was used to calculate the final copy number: http://www.scienceprimer.com/copy-number-calculator-for-realtime-pcr.

### Generation and analysis of the bulk-RNA-seq data

Total RNA was isolated from cells using RNeasy Plus Mini kit (Qiagen) according to manufacturer’s instructions. The RNA concentration and integrity was determined on the NanoDrop ND-1000 (Thermo Fisher Scientific). One thousand nanograms RNA from each sample was used as a template for preparing Illumina compatible libraries using the TruSeq RNA Library Prep Kit v2 (Illumina). Library sizes were checked using D5000 high sensitivity tape on the TapesSation 2200 (Agilent), and pooled libraries concentration were determined by Qubit 3.0 Fluorometer (Thermo Fisher Scientific). A library input of 1.8 pM with 1% PhiX (Illumina) spike-in was sequenced using the NextSeq 500 instrument (Illumina) with the NextSeq 500/550 High Output v2.5 Kit (Illumina).

RNA-Seq data were quality checked with FastQC [80] and preprocessing was carried out using TrimGalore [81] toolkit to trim low-quality bases and Illumina adapter sequences using default settings. Reads were aligned to the ENSEMBL Homo_sapiens.GRCh38.dna.primary_assembly genome using the ENSEMBL Homo_sapiens.GRCh38.100 transcript [82] annotation file with STAR [83] splice-aware RNA-seq alignment tool in paired mode allowing maximum multimapping of 3. Gene level counts were quantified using FeatureCounts [84] tool, counting unique features in non-stranded mode and retaining both gene ID, gene name, and gene biotype mapping from the ENSEMBL annotation file. Prior to differential expression analysis, count data was collapsed to donor level and genes for which mean raw count was at least 15 were kept. Normalization and differential expression were carried out with DESeq2 [85] Bioconductor [86] package, utilizing the ‘apeglm’ Bioconductor package [87] for log fold change shrinkage, in R statistical programming environment. The design formula was constructed to test for the effect of treatment while controlling for donor.

The RNA-seq data was processed using the TrimGalore toolkit [1] which employs Cutadapt [2] to trim low-quality bases and Illumina sequencing adapters from the 3′ end of the reads. Only reads that were 20nt or longer after trimming were kept for further analysis. Reads were mapped to the GRCh38v93 version of the human genome and transcriptome [3] using the STAR RNA-seq alignment tool [4]. Reads were kept for subsequent analysis if they mapped to a single genomic location. Gene counts were compiled using the HTSeq tool [5]. Only genes that had at least 10 reads in any given library were used in subsequent analysis. Normalization and differential expression was carried out using the DESeq2 [6] Bioconductor [7] package with the R statistical programming environment [8]. The false discovery rate was calculated to control for multiple hypothesis testing. GSEA [9] was performed to identify GO terms and pathways associated with altered gene expression for each of the comparisons performed.

### Statistics

For all qRT-PCR experiments, unpaired *t* tests were used. Additionally, an ordinary 1-way ANOVA with Tukey’s multiple comparison test was used to compared SARS-CoV-2 viral RNA levels between the experimental groups. All analyses were performed using GraphPad Prism 9 (La Jolla, CA).

### Biocontainment and biosafety

Biocontainment work for SARS-CoV-2 was performed with approved standard operating procedures in the Duke Regional Biocontainment Laboratory.

## Supplementary information

Supplemental Material

## Data Availability

The data that support the findings of this study are available from the corresponding authors upon request.
