# Supplementary material for: E-cigarette and food flavoring diacetyl alters airway cell morphology, inflammatory and antiviral response, and susceptibility to SARS-CoV-2
Source: Cell Death Discov. 2022 Feb 15;8:64. doi: 10.1038/s41420-022-00855-3 (PMC8847558; doi:10.1038/s41420-022-00855-3)
Supplement: Supplementary file 1 — Supplemental Material [file 41420_2022_855_MOESM1_ESM.docx]

**Supplemental Materials**

**E-cigarette and food flavoring diacetyl alters airway cell morphology, inflammatory and antiviral response, and susceptibility to SARS-CoV-2.**

Stephanie N. Langel, Francine L. Kelly, David M. Brass, Andrew E. Nagler, Dylan Carmack, Joshua Tu, Tatianna Travieso, Ria Goswami, Sallie R. Permar, Maria Blasi and Scott M. Palmer

**
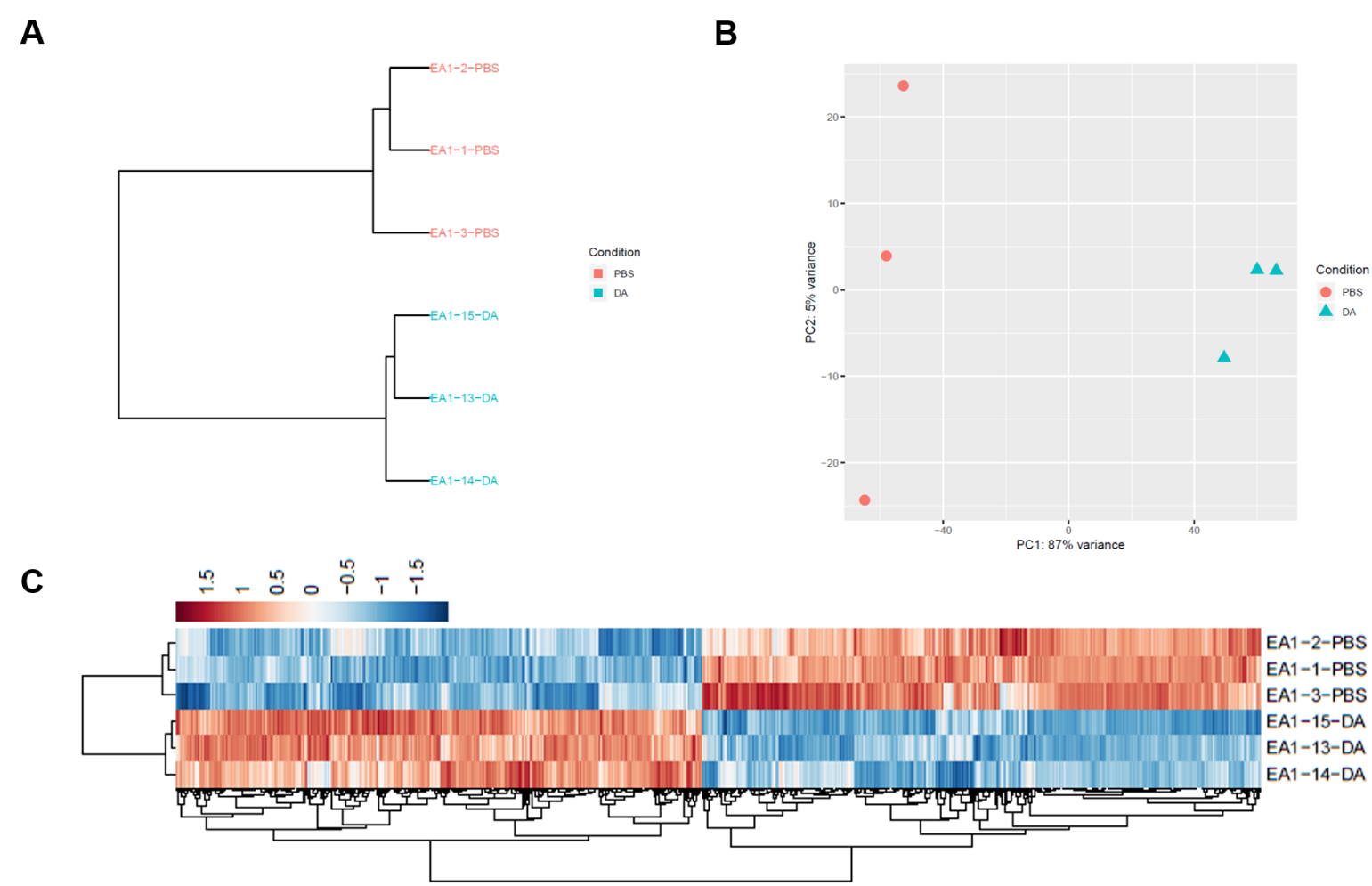
Figure S1: Diacetyl (DA) vapor exposure of normal human bronchial epithelial cells (NHBECs) drives differential transcriptomic responses.** (**A**) Hierarchical clustering of different samples (wells) based on all genes using a correlation distance with complete linkage. The sample names are colored by condition. (**B**) Principal component analysis (PCA) plot depicts clustering of PBS vehicle treated wells separate from DA vapor treated wells. (**C**) Heatmap shows expression of differentially expressed genes (FDR ≤ 5%) for each DA vapor vs PBS vehicle exposed comparison. The color scale illustrates the log_2_ fold change values shown in the heatmap.

**Table S1. Enriched gene ontology (GO) pathways with FWER p-value of ≤0.01 among downregulated genes.**

| **NAME** | **SIZE** | **ES** | **NES** | **NOM p-val** | **FDR q-val** | **FWER p-val** | **RANK AT MAX** |
| --- | --- | --- | --- | --- | --- | --- | --- |
| GO_CILIUM_ORGANIZATION | 160 | -0.79993415 | -2.8352525 | 0 | 0 | 0 | 2364 |
| GO_CILIUM_MORPHOGENESIS | 175 | -0.78368753 | -2.808809 | 0 | 0 | 0 | 2364 |
| GO_CILIARY_PART | 260 | -0.7534201 | -2.7626796 | 0 | 0 | 0 | 1911 |
| GO_CILIARY_PLASM | 73 | -0.85110605 | -2.758341 | 0 | 0 | 0 | 1499 |
| GO_CILIUM | 388 | -0.7137713 | -2.7021441 | 0 | 0 | 0 | 1911 |
| GO_MICROTUBULE_BASED_MOVEMENT | 187 | -0.7280942 | -2.6498644 | 0 | 0 | 0 | 2138 |
| GO_CELLULAR_COMPONENT_ASSEMBLY_INVOLVED_IN_MORPHOGENESIS | 207 | -0.722085 | -2.6212523 | 0 | 0 | 0 | 2364 |
| GO_CILIARY_BASAL_BODY | 73 | -0.81247824 | -2.6161053 | 0 | 0 | 0 | 1761 |
| GO_PRIMARY_CILIUM | 174 | -0.7138895 | -2.5855541 | 0 | 0 | 0 | 1911 |
| GO_CELL_PROJECTION_ASSEMBLY | 230 | -0.6878841 | -2.5348134 | 0 | 0 | 0 | 2138 |
| GO_MICROTUBULE_BUNDLE_FORMATION | 54 | -0.8197918 | -2.5289617 | 0 | 0 | 0 | 1083 |
| GO_MOTILE_CILIUM | 87 | -0.76097673 | -2.5105822 | 0 | 0 | 0 | 1740 |
| GO_INTRACILIARY_TRANSPORT_PARTICLE | 29 | -0.9072645 | -2.4906635 | 0 | 0 | 0 | 1843 |
| GO_MICROTUBULE_MOTOR_ACTIVITY | 72 | -0.76743793 | -2.4813232 | 0 | 0 | 0 | 2529 |
| GO_PROTEIN_TRANSPORT_ALONG_MICROTUBULE | 26 | -0.9083011 | -2.4596906 | 0 | 0 | 0 | 1495 |
| GO_AXONEME_ASSEMBLY | 33 | -0.86312264 | -2.4563868 | 0 | 0 | 0 | 2363 |
| GO_DYNEIN_COMPLEX | 41 | -0.84373736 | -2.451341 | 0 | 0 | 0 | 1204 |
| GO_CILIUM_MOVEMENT | 30 | -0.892315 | -2.4196167 | 0 | 0 | 0 | 2138 |
| GO_CILIARY_TIP | 42 | -0.81657153 | -2.4179215 | 0 | 0 | 0 | 2180 |
| GO_SPECIFICATION_OF_SYMMETRY | 95 | -0.7235856 | -2.407554 | 0 | 0 | 0 | 1895 |
| GO_AXONEME_PART | 20 | -0.9363061 | -2.3932087 | 0 | 0 | 0 | 1151 |
| GO_NONMOTILE_PRIMARY_CILIUM | 112 | -0.6916825 | -2.3846126 | 0 | 0 | 0 | 1473 |
| GO_MICROTUBULE_ASSOCIATED_COMPLEX | 136 | -0.6791725 | -2.3821623 | 0 | 0 | 0 | 2529 |
| GO_PROTEIN_COMPLEX_LOCALIZATION | 50 | -0.7815072 | -2.375584 | 0 | 0 | 0 | 1495 |
| GO_MICROTUBULE_ORGANIZING_CENTER_PART | 132 | -0.683075 | -2.3713071 | 0 | 0 | 0 | 3564 |
| GO_CENTRIOLE | 91 | -0.7104294 | -2.3593984 | 0 | 0 | 0 | 3564 |
| GO_SMOOTHENED_SIGNALING_PATHWAY | 65 | -0.74410063 | -2.3567252 | 0 | 0 | 0 | 2574 |
| GO_CILIARY_TRANSITION_ZONE | 22 | -0.89642686 | -2.3487153 | 0 | 0 | 0 | 1911 |
| GO_MICROTUBULE_BASED_PROCESS | 460 | -0.6085874 | -2.3112705 | 0 | 0 | 0 | 2550 |
| GO_SPERM_FLAGELLUM | 48 | -0.7545017 | -2.282854 | 0 | 0 | 0 | 1666 |
| GO_INTRACILIARY_TRANSPORT_PARTICLE_B | 18 | -0.8952823 | -2.2576864 | 0 | 0 | 0 | 1834 |
| GO_AXONEMAL_DYNEIN_COMPLEX_ASSEMBLY | 16 | -0.91866493 | -2.2442555 | 0 | 0 | 0 | 786 |
| GO_CENTROSOME | 446 | -0.5839033 | -2.2381663 | 0 | 0 | 0 | 3656 |
| GO_SPERM_MOTILITY | 34 | -0.7841684 | -2.2209575 | 0 | 0 | 0 | 2327 |
| GO_MOTOR_ACTIVITY | 116 | -0.63588 | -2.1992662 | 0 | 0 | 0 | 2529 |
| GO_MICROTUBULE | 365 | -0.57722616 | -2.1815147 | 0 | 0 | 0 | 3130 |
| GO_MICROTUBULE_CYTOSKELETON_ORGANIZATION | 302 | -0.5845332 | -2.172768 | 0 | 0 | 0 | 3840 |
| GO_ORGANELLE_ASSEMBLY | 433 | -0.5732703 | -2.1714826 | 0 | 0 | 0 | 2375 |
| GO_REGULATION_OF_SMOOTHENED_SIGNALING_ PATHWAY | 56 | -0.69079113 | -2.1489303 | 0 | 0 | 0 | 2734 |
| GO_EPITHELIAL_CILIUM_MOVEMENT | 17 | -0.8651223 | -2.1070154 | 0 | 1.58E-05 | 0.001 | 2138 |
| GO_NONMOTILE_PRIMARY_CILIUM_ASSEMBLY | 22 | -0.8225812 | -2.097683 | 0 | 1.54E-05 | 0.001 | 2364 |
| GO_ESTABLISHMENT_OF_LOCALIZATION_BY_MOVEMENT_ALONG_MICROTUBULE | 89 | -0.63095546 | -2.0975559 | 0 | 1.50E-05 | 0.001 | 2074 |
| GO_PEPTIDYL_GLUTAMIC_ACID_MODIFICATION | 24 | -0.78339267 | -2.0409365 | 0 | 1.77E-04 | 0.012 | 2138 |
| GO_VENTRICULAR_SYSTEM_DEVELOPMENT | 25 | -0.75638825 | -2.0398116 | 0 | 1.72E-04 | 0.012 | 2352 |
| GO_PHOTORECEPTOR_CONNECTING_CILIUM | 29 | -0.7342037 | -2.0300179 | 0 | 1.97E-04 | 0.013 | 1473 |

**Table S2. Cilia structural and motility genes associated with significantly downregulated gene ontology (GO) pathways.**

| **Gene/Gene group** | **Function** | **Gene name** | **Log_2_ fold change (DA/PBS)** | **Adjusted P-value** | **Average PBS** | **Average DA** | **Number of occurrences in top 10 down regulated pathways** |
| --- | --- | --- | --- | --- | --- | --- | --- |
| Transcription factors | Regulate transcription of genes that control ciliogenesis | FOXJ1 | -1.96 | 1.21E-39 | 43670.60 | 11209.22 | 4/10 |
|  |  | RFX2 | -2.25 | 1.06E-68 | 9298.59 | 1949.07 | 4/10 |
|  |  | RFX3 | -1.87 | 1.58E-63 | 7846.62 | 2149.20 | 5/10 |
| Cyclin-O | Required for generation of multiciliated cells | CCNO | -1.41 | 9.92E-44 | 16470.21 | 6207.31 | 4/10 |
| Radial spoke/ Radial spoke head | Provides structure through interacting with central apparatus, required for motile cilia function | DNAJB13 | -2.19 | 3.73E-63 | 1984.37 | 435.73 | 0/10 |
|  |  | RSPH3 | -1.39 | 1.52E-37 | 8550.91 | 3265.05 | 0/10 |
|  |  | RSPH1 | -2.75 | 6.06E-67 | 35511.28 | 5261.57 | 6/10 |
|  |  | RSPH4A | -2.79 | 2.79E-65 | 16303.85 | 2363.80 | 7/10 |
|  |  | RSPH9 | -3.02 | 5.97E-23 | 5189.44 | 638.99 | 7/10 |
| Dynein Axonemal Intermediate Chains/ Outer dynein arm complex | Part of dynein complex in respiratory cilia, serves as anchor for other complexes involved in motility | DNAI1 | -2.86 | 1.35E-85 | 16946.29 | 2331.35 | 8/10 |
|  |  | DNAI2 | -3.48 | 1.26E-99 | 10371.82 | 930.20 | 8/10 |
|  |  | DNAH11 | -2.20 | 5.97E-102 | 9345.96 | 2027.56 | 2/10 |
|  |  | DNAH5 | -2.47 | 1.57E-109 | 17148.63 | 3105.55 | 8/10 |
|  |  | DNAH6 | -2.84 | 9.38E-121 | 11112.18 | 1551.99 | 3/10 |
|  |  | DNAH9 | -3.86 | 3.52E-179 | 36343.99 | 2503.39 | 3/10 |
|  |  | CCDC114 | -1.85 | 1.76E-43 | 16330.32 | 4534.63 | 7/10 |
|  |  | CCDC151 | -3.16 | 1.87E-101 | 4940.63 | 553.78 | 8/10 |
| Dynein Axonemal Heavy Chains/Inner dynein arm complex | Force generating proteins required for motile cilia function | DNAH10 | -3.56 | 1.54E-256 | 11991.63 | 1017.02 | 2/10 |
|  |  | DNAH1 | -1.92 | 9.04E-56 | 4997.91 | 2149.20 | 8/10 |
|  |  | DNAH7 | -2.32 | 5.44E-88 | 9618.52 | 1931.18 | 8/10 |
|  |  | CCDC39 | -1.51 | 4.40E-10 | 143.24 | 50.45 | 7/10 |
|  |  | CCDC40 | -1.95 | 2.70E-62 | 15980.86 | 4127.90 | 8/10 |
| Intraflagellar Transport | Maintenance and formation of cilia | IFT122 | -2.15 | 2.14E-75 | 9333.10 | 2102.09 | 6/10 |
|  |  | IFT172 | -2.26 | 1.40E-82 | 17344.03 | 3624.14 | 7/10 |
|  |  | IFT140 | -1.78 | 1.83E-68 | 14986.99 | 4362.41 | 7/10 |
|  |  | IFT80 | -1.42 | 6.12E-35 | 2801.09 | 1050.13 | 6/10 |
|  |  | IFT81 | -1.49 | 1.25E-32 | 5024.39 | 1782.99 | 7/10 |
| Kinesins | Required for motile cilia function, regulates length of cilia | KIF17 | -3.15 | 4.62E-101 | 1134.28 | 127.28 | 6/10 |
|  |  | KIF19 | -2.73 | 9.83E-89 | 10455.15 | 1573.34 | 4/10 |
|  |  | KIF24 | -2.39 | 3.93E-88 | 4473.63 | 852.48 | 5/10 |
|  |  | KIF27 | -1.25 | 1.87E-03 | 2922.07 | 1230.89 | 6/10 |
| Axonemal Central Pair Apparatus Protein | Ciliary motility | HYDIN | -2.84 | 5.45E-118 | 10823.25 | 1507.00 | 8/10 |
| α-tubulin | Microbutuble structure | TUBA1A | -2.65 | 3.46E-90 | 61254.05 | 9727.76 | 0/10 |

**Table S3. Significantly enriched GO pathways among upregulated genes.**

| **NAME** | **SIZE** | **ES** | **NES** | **NOM p-val** | **FDR q-val** | **FWER p-val** | **Genes with adjusted P-value <0.001 and Log2 fold change > 1.5** |
| --- | --- | --- | --- | --- | --- | --- | --- |
| GO_LIPOPOLYSACCHARIDE_MEDIATED_SIGNALING_PATHWAY | 30 | 0.6989776 | 2.177455 | 0 | 0.020024627 | 0.01 | PTPN22, LY96, CD14, IL1B |
| GO_POSITIVE_REGULATION_OF_EPITHELIAL_CELL_MIGRATION | 93 | 0.5372326 | 2.1680748 | 0 | 0.012068383 | 0.012 | HAS2, ITGA2, TEK, FGF2, KDR, INSL3, WNT7A, PTGS2 |
| GO_CELLULAR_RESPONSE_TO_MECHANICAL_STIMULUS | 74 | 0.56645846 | 2.1549094 | 0 | 0.009354376 | 0.014 | IL13, PDE2A, IGF2, TNFSF14, ATP1A2, ANKRD1, IL1B, BMP6, PTGS2 |

**
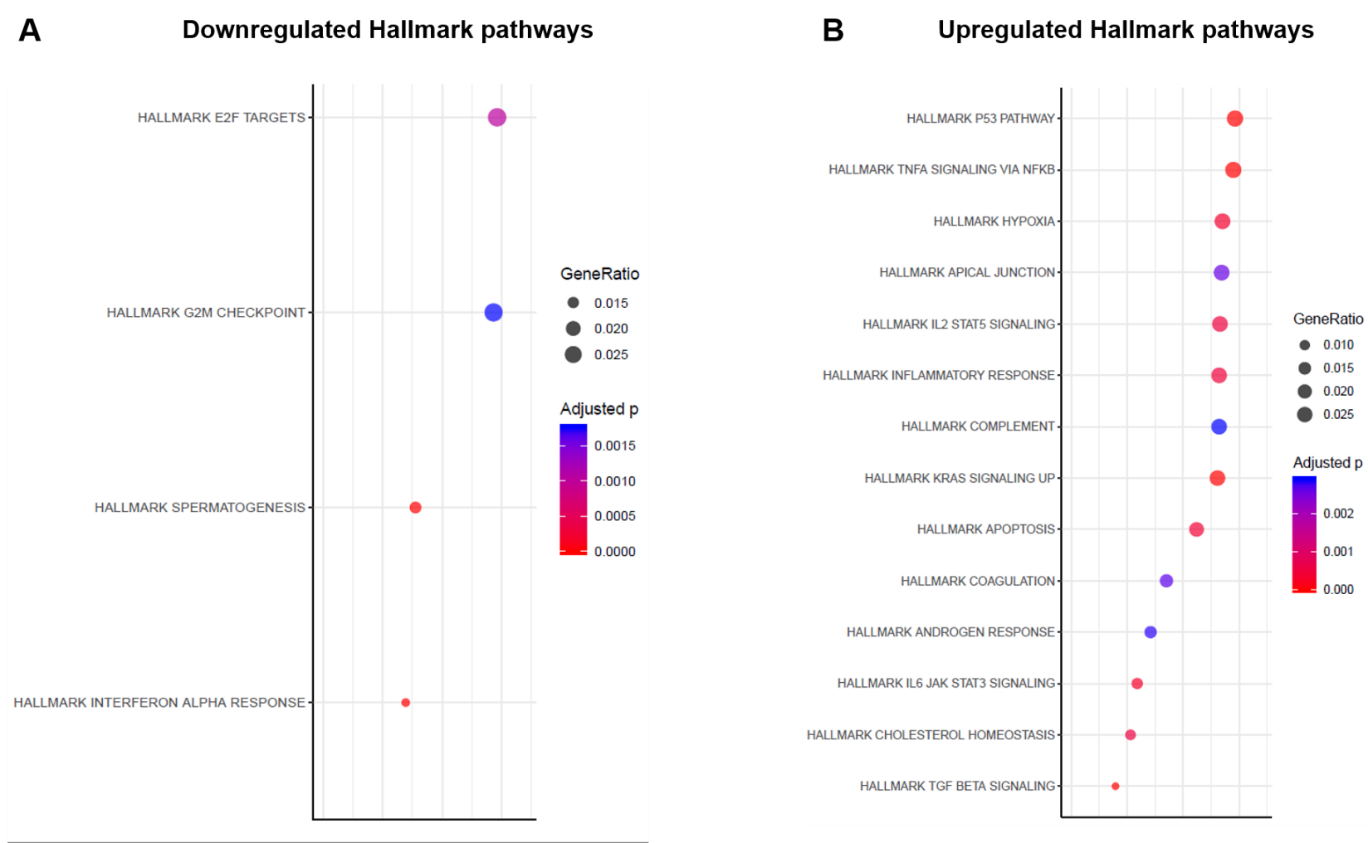
**

**Figure S2. Hallmark pathways associated with interferon responses and DNA damage repair are downregulated in DA exposed normal human bronchial epithelial cells while pathways associated with sterile inflammation are upregulated.** (**A**) Down- and (**B**) upregulated Hallmark pathways with an FWER adjusted p value < 0.015 were reported. Dot size represents gene ratio and color schema represents FWER adjusted p values.

**Table S4. Significantly enriched Hallmark pathways among upregulated genes.**

| **NAME** | **SIZE** | **ES** | **NES** | **NOM p-val** | **FDR q-val** | **FWER p-val** |
| --- | --- | --- | --- | --- | --- | --- |
| HALLMARK_TNFA_SIGNALING_VIA_NFKB | 194 | 0.644173 | 2.829616 | 0 | 0 | 0 |
| HALLMARK_KRAS_SIGNALING_UP | 175 | 0.533082 | 2.389338 | 0 | 0 | 0 |
| HALLMARK_P53_PATHWAY | 196 | 0.539526 | 2.380517 | 0 | 0 | 0 |
| HALLMARK_TGF_BETA_SIGNALING | 53 | 0.654998 | 2.374477 | 0 | 0 | 0 |
| HALLMARK_INFLAMMATORY_RESPONSE | 177 | 0.46263 | 2.041284 | 0 | 4.67E-04 | 0.001 |
| HALLMARK_APOPTOSIS | 150 | 0.469152 | 2.018132 | 0 | 3.89E-04 | 0.001 |
| HALLMARK_HYPOXIA | 181 | 0.446414 | 1.975552 | 0 | 3.33E-04 | 0.001 |
| HALLMARK_IL6_JAK_STAT3_SIGNALING | 79 | 0.499977 | 1.962443 | 0 | 2.92E-04 | 0.001 |
| HALLMARK_CHOLESTEROL_HOMEOSTASIS | 71 | 0.501458 | 1.950917 | 0 | 5.70E-04 | 0.002 |
| HALLMARK_IL2_STAT5_SIGNALING | 178 | 0.440024 | 1.936171 | 0 | 5.13E-04 | 0.002 |
| HALLMARK_ANDROGEN_RESPONSE | 95 | 0.442639 | 1.796753 | 0 | 0.002829 | 0.009 |
| HALLMARK_APICAL_JUNCTION | 180 | 0.412476 | 1.791653 | 0 | 0.002594 | 0.009 |
| HALLMARK_COMPLEMENT | 177 | 0.396347 | 1.75461 | 0 | 0.002881 | 0.011 |
| HALLMARK_COAGULATION | 114 | 0.418533 | 1.750745 | 0 | 0.002675 | 0.011 |
| HALLMARK_EPITHELIAL_MESENCHYMAL_TRANSITION | 182 | 0.351413 | 1.579242 | 0 | 0.010699 | 0.045 |

**Table S5. Significantly downregulated interferon stimulated genes after diacetyl exposure in normal human bronchial epithelial cells.**

| **Gene** | **Name** | **Function** | **Log_2_ fold change** | **Adjusted**  **p-value** | **Average PBS** | **Average DA** |
| --- | --- | --- | --- | --- | --- | --- |
| ZBP1 | Z-DNA-Binding Protein 1 | Innate immune sensor of both RNA (influenza virus) and DNA (murine cytomegalovirus) viruses | -4.985939986 | 1.74617E-09 | 344.2549036 | 10.85486478 |
| CXCL11 | C-X-C motif chemokine 11 | Chemotactic factor, cell recruitment/migration | -4.236313715 | 2.90849E-08 | 615.2025627 | 32.61034851 |
| CXCL10 | C-X-C motif chemokine 10 | Chemotactic factor, cell recruitment/migration | -4.217054166 | 3.72117E-06 | 2386.538961 | 128.2929262 |
| RSAD2 | Radical SAM domain-containing 2 (also known as Viperin) | Inhibits RNA (influenza, Zika) and DNA (HIV-1) viruses | -4.06988 | 2.45E-16 | 6066.522 | 361.1785 |
| CXCL9 | C-X-C motif chemokine 9 | Chemotactic factor, cell recruitment/migration | -3.689616755 | 1.72424E-12 | 61.94073147 | 4.786680896 |
| IFI6 | Interferon Alpha Inducible Protein 6 | Blocks flavivirus replication | -3.18676 | 8.82E-11 | 16393.16 | 1800.266 |
| IFI44L | Interferon-Induced protein 44-Like | Prevents viral infection (respiratory syncytial virus, hepatitis C virus) | -2.943136222 | 9.58603E-06 | 4582.270511 | 595.7631328 |
| CMPK2 | Cytidylate Monophosphase Kinase 2 | Associated with type 1 interferon–induced HIV restriction in humans | -2.72669846 | 5.67537E-09 | 2724.474495 | 411.5221287 |
| IFIT1 | Interferon Induced Protein With Tetratricopeptide Repeats 1 | Antiviral protein that recognizes 5′-triphosphate RNA | -2.66176 | 2.03E-06 | 5073.959 | 801.7617 |
| SAMHD1 | SAM and HD Domain Containing Deoxynucleoside Triphosphate Triphosphohydrolase 1 | Depletes dNTPs and restricts viral replication of DNA viruses including HIV-1 | -2.59064 | 3.12E-77 | 24662.83 | 4094.119 |
| MX2 | Interferon-Induced GTP-binding protein Mx2 | Inhibits several different viruses (HIV-1, herpesviruses) by blocking early steps of the viral replication cycle | -2.55722 | 2.61E-09 | 8395.852 | 1426.412 |
| IFI27 | Interferon Alpha-Inducible protein 27 | Induces apoptosis in virally infected cells | -2.55224 | 1.05E-09 | 17887.83 | 3049.625 |
| ISG15 | Interferon-Stimulated Gene 15 | inhibits influenza A virus replication and thus contributes to the antiviral action of IFN-β | -2.29635 | 1.23E-08 | 11739.25 | 2389.774 |
| EPSTI1 | Epithelial-stromal interaction 1 | Inhibits hepatitis C virus replication | -2.225928575 | 4.5726E-08 | 1712.445902 | 365.9651665 |
| CCL5 | Chemokine ligand 5 | Blocks viral-induced apoptosis | -2.050545412 | 0.000204203 | 2313.399819 | 558.4283036 |
| IFIT3 | Interferon Induced Protein With Tetratricopeptide Repeats 3 | IFN-induced antiviral protein which acts as an inhibitor of cellular as well as viral processes(viral replication) | -1.92377 | 5.23E-08 | 7025.918 | 1851.667 |
| MX1 | MX Dynamin Like GTPase 1 | Inhibits Influenza Virus by Interfering with Functional Viral Ribonucleoprotein Complex Assembly | -1.85168 | 2.58E-07 | 12001.32 | 3325.088 |
| IFITM1 | Interferon Induced Transmembrane Protein 1 | Modulates viral entry into cells (including coronavirus) | -1.76367 | 5.28E-22 | 3561.903 | 1048.631 |
| XAF1 | X-linked inhibitor of apoptosis (XIAP)-associated factor 1 | Enhances cellular antiviral responses | -1.76149 | 0.000431 | 4280.345 | 1262.396 |
| CCL2/ MCP-1 | Monocyte chemotactic protein-1 | Chemotactic factor, cell recruitment/migration | -1.703386353 | 6.53801E-13 | 169.4921257 | 51.97096519 |
| BST2 | Bone Marrow Stromal Cell Antigen 2 (also known as tetherin) | Blocks the egress of enveloped viruses from infected cell | -1.63527 | 4.52E-06 | 3517.758 | 1132.277 |


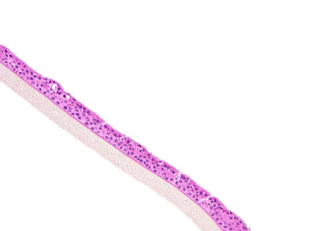

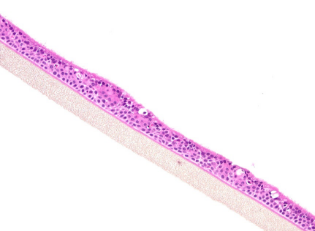

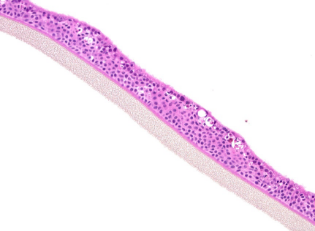

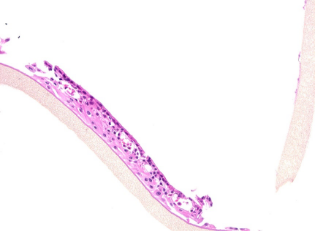

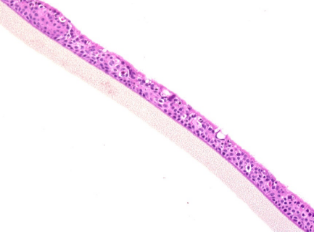

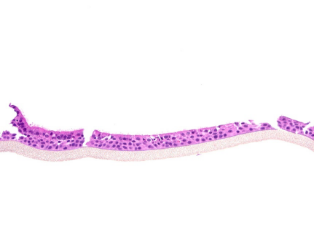

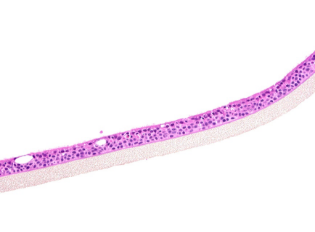

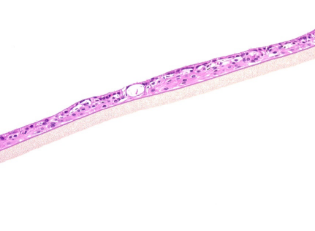


PBS no virus

PBS with virus


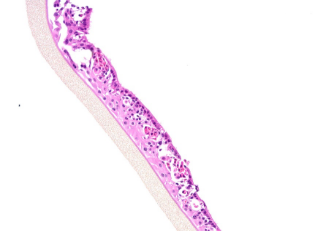

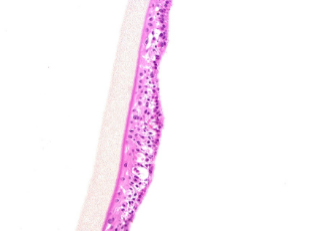

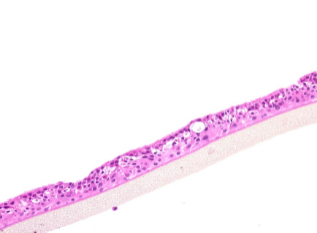

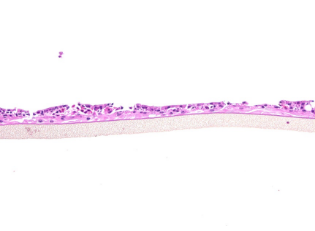


DA with virus

DA no virus

**Figure S3. SARS-CoV-2 infection does not further exacerbate cellular dysplasia or cilia loss in DA vapor exposed normal human bronchial epithelial cells (NHBECs).** Pathological assessment after hematoxylin and eosin (H&E) staining of PBS vehicle/no virus, DA vapor/no virus, PBS vehicle/with virus and DA vapor/with virus.
